# Clinical severity of aneurysmal subarachnoid hemorrhage over time: systematic review

**DOI:** 10.1007/s10143-024-02467-0

**Published:** 2024-06-05

**Authors:** Manou Overstijns, Amir El Rahal, Johannes Goldberg, Roland Rölz, Andreas Raabe, Karin Bischoff, Nicolas Noël Neidert, Jürgen Beck, Christian Fung

**Affiliations:** 1 Department of Neurosurgery, Medical Center University of Freiburg, Freiburg, Germany; 2 Faculty of Medicine of Geneva, University of Geneva, Geneva, Switzerland; 3 Department of NeurosurgeryInselspitalBern University Hospital, University of Bern, Bern, Switzerland; 4 Clinical Trials UnitUniversity Medical Center FreiburgFaculty of Medicine, , University of Freiburg, Freiburg, Germany; 5 Berta-Ottenstein-Programme for Clinician Scientists Medical Center, University of Freiburg, Freiburg, Germany

**Keywords:** Subarachnoid hemorrhage, Poor grade, Incidence, Trend, Outcome

## Abstract

The incidence of aneurysmal subarachnoid hemorrhage (aSAH) is well studied. Yet, little is known about the trend of aSAH severity. This systematic review aims to analyze the distribution of aSAH severity over time. We performed a systematic review of the literature according to the PRISMA-P guidelines. We included studies from January 1968 up to December 2022. Studies were included if they either reported the severity of aSAH as single increments of the corresponding 5-point scale or as a binary measure (good grade 1-3, poor grade 4-5) on the Hunt and Hess (HH) or World Federation of Neurosurgical Societies (WFNS) scale. Studies with fewer than 50 patients, (systematic) reviews, and studies including non-aSAH patients were excluded. A total of 2465 publications were identified, of which 214 met the inclusion and exclusion criteria. In total, 102,845 patients with an aSAH were included. Over the last five decades the number of good-grade HH (0.741 fold, *p* = 0.004) and WFNS (0.749 fold, *p* < 0.001) has decreased. Vice versa, an increase in number of poor grade HH (2.427 fold, *p* = 0.004), WFNS (2.289 fold, *p* < 0.001), as well as HH grade 5 (6.737 fold, *p* = 0.010), WFNS grade 4 (1.235 fold, *p* = 0.008) and WFNS grade 5 (8.322 fold, *p* = 0.031) was observed. This systematic review shows a worldwide 2-3 fold increase of poor grade aSAH patients and an 6-8 fold increase of grade 5 patients, over the last 50 years. Whether this evolution is due to more severe hemorrhage, improvements in neuro-intensive care and prehospital management, or to a change in grading behavior is unknown. This study strongly emphasizes the necessity for an improved grading system to differentiate grade 4 and grade 5 patients for meaningful clinical decision- making.

## Introduction

The global incidence of aneurysmal subarachnoid haemorrhage (aSAH) declined by 40% between 1980 and 2010, probably explained by a decrease in hypertension, smoking prevalence and the rise in screening and preventive repair of unruptured intracranial aneurysms [1]. The identification of these trends is vital for the formulation of effective prevention strategies and for mitigating the disease’s overall burden [1]. Critical to the management of aSAH is the clinical assessment of disease severity at the time of patient admission, typically evaluated using the Hunt and Hess (HH) or the World Federation of Neurosurgical Societies (WFNS) scales [2, 3]. By convention, the grades 1–3 are deemed as good grade aSAH and grades 4–5 as poor grade aSAH [4]. While the incidence of aSAH over time is well studied, the distribution of the different grades of aSAH severity and its development over time is currently unclear.

Previous smaller retrospective studies indicated no significant change in the distribution of aSAH grades over time [5, 6]. In contrast, a systematic review showed an increase of the amount of grade 5 aSAH patients over time [7]. The observed trend in the severity of aSAH over time is multifaceted, potentially reflecting advancements in emergency medical services, changes in hospital referral patterns, improvements in diagnostic capabilities, and possibly variations in grading practices among clinicians. Understanding these trends is crucial for comprehending the evolving landscape of aSAH management, improving the accuracy of patient prognosis, and optimizing therapeutic strategies to enhance outcomes for affected individuals. Due to advances in neurocritical care, surgical techniques, and endovascular treatments, poor grade patients can achieve a favourable outcomes in up to 50% of cases [7]. It is paramount to ascertain whether current severity grading systems adequately reflect these positive changes. The evolution in patient management and care highlights the need for a critical examination of whether severity grades continue to serve as accurate prognostic markers. This aspect is particularly relevant for guiding treatment strategies and for the ongoing refinement of prognostic models, ensuring they align with contemporary clinical realities. The trend in aSAH severity and its implications is a complex issue, influenced by multiple factors including improvements in pre-hospital care, diagnostic precision, and possibly shifts in grading practices. This systematic review aims to close this data gap and analyze the distribution of aSAH severity at admission, using the HH and the WFNS grading system covering the last 5 decades.

## Materials and methods

### Reporting guideline

We adhered to the Preferred Reporting Items for System- atic reviews and Meta-Analyses (PRISMA-P) protocol for identifying, screening and eligibility of studies to conduct the present research work.

### Information sources and search strategy

We searched for original research that reported data on the severity of aSAH between 1 January 1968 (creation of the Hunt and Hess score) and 7 December 2022. Published studies were identified from two electronic databases: Medline (via Pubmed) and Embase. The search strategy was based on combinations of medical subject headings (MeSH) and Emtree keywords and was restricted to original English articles that report the range of aSAH severity as graded either on the WFNS scale or the HH scale at admission. The search strategy used in Medline (via PubMed) and Embase is presented in an additional file.

### Eligibility criteria

Studies were included if (1) they confirmed the aSAH using neuroimaging (either CT- angiography, MRI-angiography or digital subtraction angiography); (2) they either report the severity of aSAH at patient admission as a single increment of the respective 5-point HH or WFNS scale or (3) the severity is dichotomized in HH or WFNS good grade (1–3) and poor grade (4–5).

Studies were excluded if they: (1) were not written in English; (2) had less than 50 patients included; (3) were case reports; (4) were (systematic) reviews or meta-analyses.

The following data were extracted: Author’s name, publication country, onset and end of study year, study design, number of patients, HH grade and WFNS Grade at admission of the patient. Good grade was defined as grade 1 to 3, and poor grade was defined as grade 4 to 5. If the complete HH/WFNS grade was described, the sum of grades 1 to 3 and 4 to 5 was calculated and subsequently included in the good and poor grade groups.

The primary outcome was the trend of good and poor grades according to the HH and the WFNS grading system. Secondary outcomes included the effect of the study country on aSAH grading and the effect of study midyear on the number of patients per study, to analyze the heterogeneity.

### Study selection

After removing duplicates, each reference was screened by two reviewers (M.O. and A.E.R.) independently using the Covidence® online software [8]. Covidence is a web-based collaboration software platform that streamlines the production of systematic and other literature reviews. Irrelevant articles were excluded based on title and abstract. Two Authors (M.O. and A.E.R.) independently extracted data using again the Covidence software. The files were cross-checked and verified against the source material. Discrepancies were analyzed and solved by a third author (C.F.).

### Quality assessment

Studies were screened independently for bias by two authors (M.O and A.E.R) using the Covidence program. Following the methodological index tool for non-randomized studies, the studies were assessed on bias regarding selection bias, detection bias, attrition bias, reporting bias and other biases [9, 10]. A thorough assessment of selective non-reporting or underreporting of results in studies was performed by two authors (M.O and A.E.R.) Studies that were assessed to have a high risk of bias were independently analyzed by a third author (C.F.). If the high bias risk resulted in unacceptably low quality of evidence, the study was excluded in concordance with the three authors (M.O., A.E.R. and C.F.).

### Statistical analysis

Statistical analyses were performed using SPSS Statistics (IBM, Version 29). To estimate the temporal trend of patients with good and poor grades according to the Hunt and Hess (HH) and World Federation of Neurological Surgeons (WFNS) scales, we calculated the midyear (midpoint year) for each study. This was achieved by first determining the sum of the beginning and end years of patient inclusion, and then dividing this sum by 2. Subsequently, studies were grouped by decade based on their midyear to facilitate analysis of trends over time. Since the WFNS was published in 1988, there is no data on WFNS grading in the decade 1970–1980. Moreover, since only 1 study described the HH good and poor grade prevalence in the study decade 1970–1980, no confidence interval can be determined in this decade. Similarly, no studies described the number of patients per HH grade during the study decade 1970–1980. While our review encompassed all studies up to December 2022, it is important to note that none of the studies included had a midyear later than 2020, resulting in the last decade being 2011 until 2020.

To determine if the study decade has a significant association with the HH or WFNS grade in good or poor grade aSAH patients, an analysis of variance (ANOVA) test was performed. Subsequently, an ANOVA test was performed for every single HH and WFNS grade to determine the trend per grade over time. A confidence interval of 95% was considered significant (*p* < 0.05). A control ANOVA analysis was performed, were the number of patients per study was weighted to analyze the heterogeneity between the included studies. A Levene test for equality of variance was performed, and if the p-value was > 0.05, equal variance for the ANOVA test was assumed. When equality of variance cannot be assumed (i.e. Levene test showed a p-value of < 0.05), a Welch test was performed to assess the means of the different groups. Following the ANOVA, Tukey’s Honest Significant Difference test was utilized for post-hoc comparisons to accurately identify which specific decades differed significantly in the grading of aSAH severity. A chi-square test was used to analyze the effect of the country of the study and the midyear of the study (categorized in decades), and the proportion of HH and WFNS good and poor grades. To investigate the relationship between the study midyear and the number of patients included in each study, we employed Analysis of Variance (ANOVA). This statistical method was chosen to analyze the differences in the number of patients across various midyears, treated as categorical variables representing different decades of study.

## Results

### Study identification and selection

**Fig. 1:**
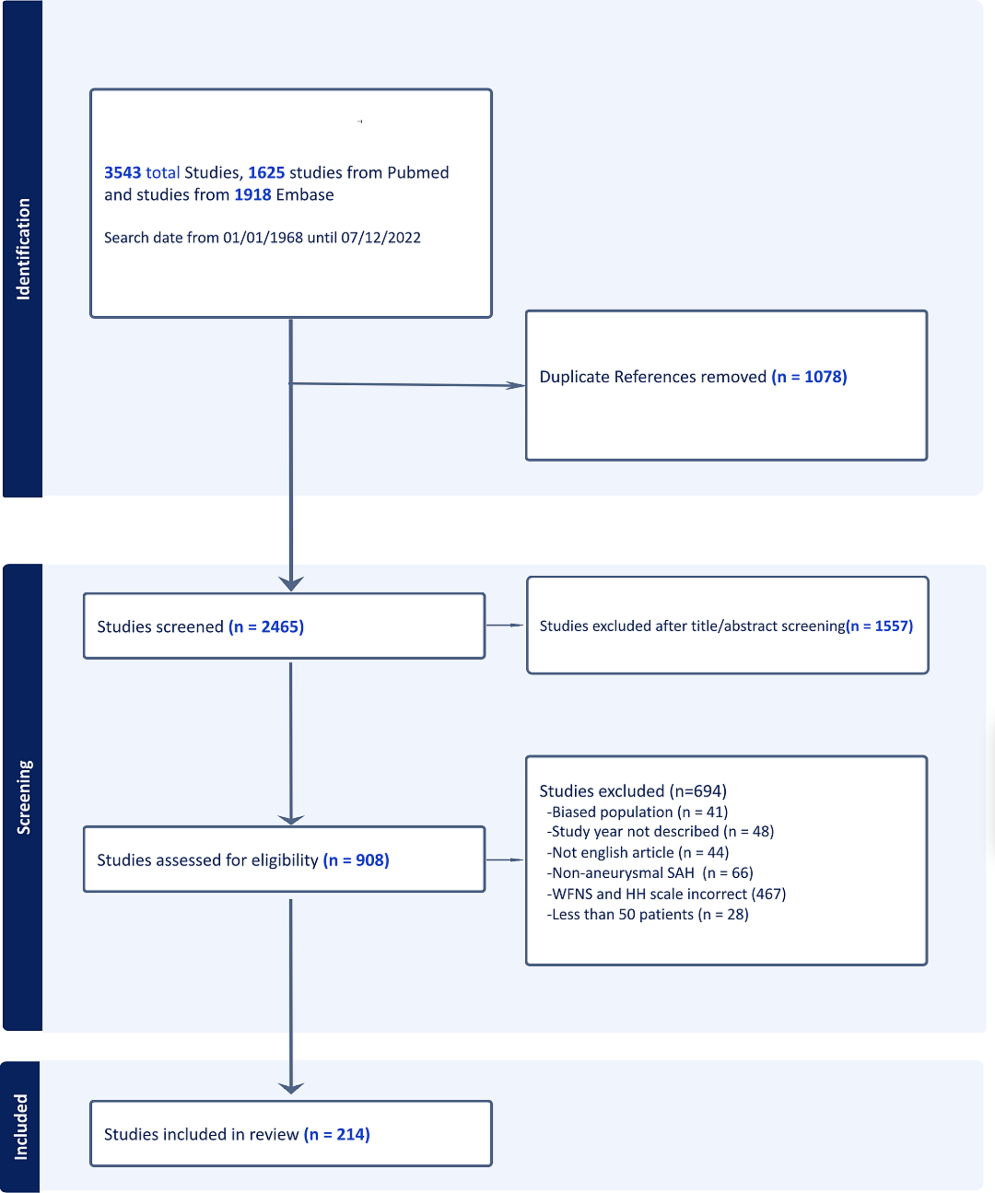
PRISMA-P flow chart for included studies using the Covidence Cochrane collaboration tool program®. In total, 3543 studies were identified, and after applying the inclusion and exclusion criteria, 214 studies were included in this systematic review. Two authors evaluated all articles, and a third author evaluated discrepancies

**Fig. 2:**
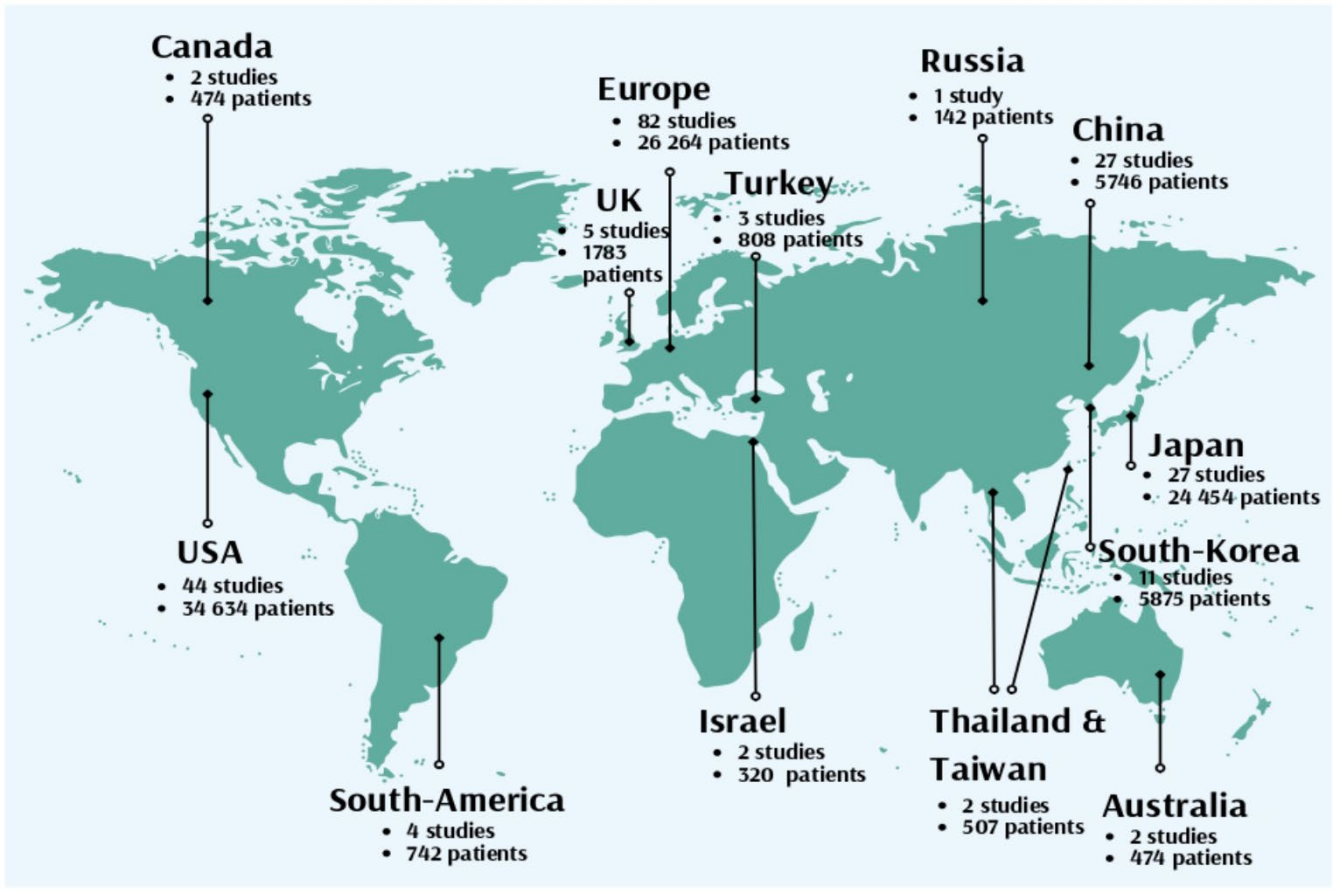
Of the 214 included studies worldwide, the majority originated in Europe (38.3%), followed by the USA (20.6%), China (12.6%) and Japan (12.6%). No significant associations were identified between the study country and HH good or poor grade, WFNS good or poor grade, or the number of studies per study decade

The literature search yielded 3543 publications (1918 from Embase and 1625 from Medline). After removing 1078 duplicates, we screened the remaining 2465 studies on title and abstract. The screening excluded 1557 utilizing the inclusion and exclusion criteria. A full-text review was performed on the remaining 908 studies. In total, 41 studies were excluded due to an unacceptable high risk of bias and low quality of evidence. Furthermore, 467 articles were removed because the HH/WFNS grades were incompletely reported, 28 studies were removed because they included less than 50 patients. Additionally, 66 studies with non-aneurysmal SAH were removed, 44 non-English studies were removed and 48 studies were removed because the study year was not described. In total, 214 studies over all continents were included in this systematic review (Fig. 1)(Fig. 2).

### Patient cohort

A total of 102.845 patients were included in the systematic review, with the midyear period ranging from 1977 to 2020. The studies were mainly retrospective (*n* = 163, 76.2%), with a smaller number of prospective studies (*n* = 49, 22.9%) and only two randomized controlled trials (0.9%).

The complete HH grade (grade 1 to 5) was described in 65 studies (30.4%), whereas the HH grade ind 40 studies (18.7%) were dichotomized in good grade and poor grade patients. Similarly, the full WFNS scale (grade 1 to 5) was described in 79 studies (36.9%) whereas the WFNS grade in 40 studies (18.7%) were dichotomized in good grade and poor grade patients. Of the 214 studies, 10 studies (4.7%) described both the HH and WFNS grade.

### Change in the hunt and hess grade

**Fig. 3:**
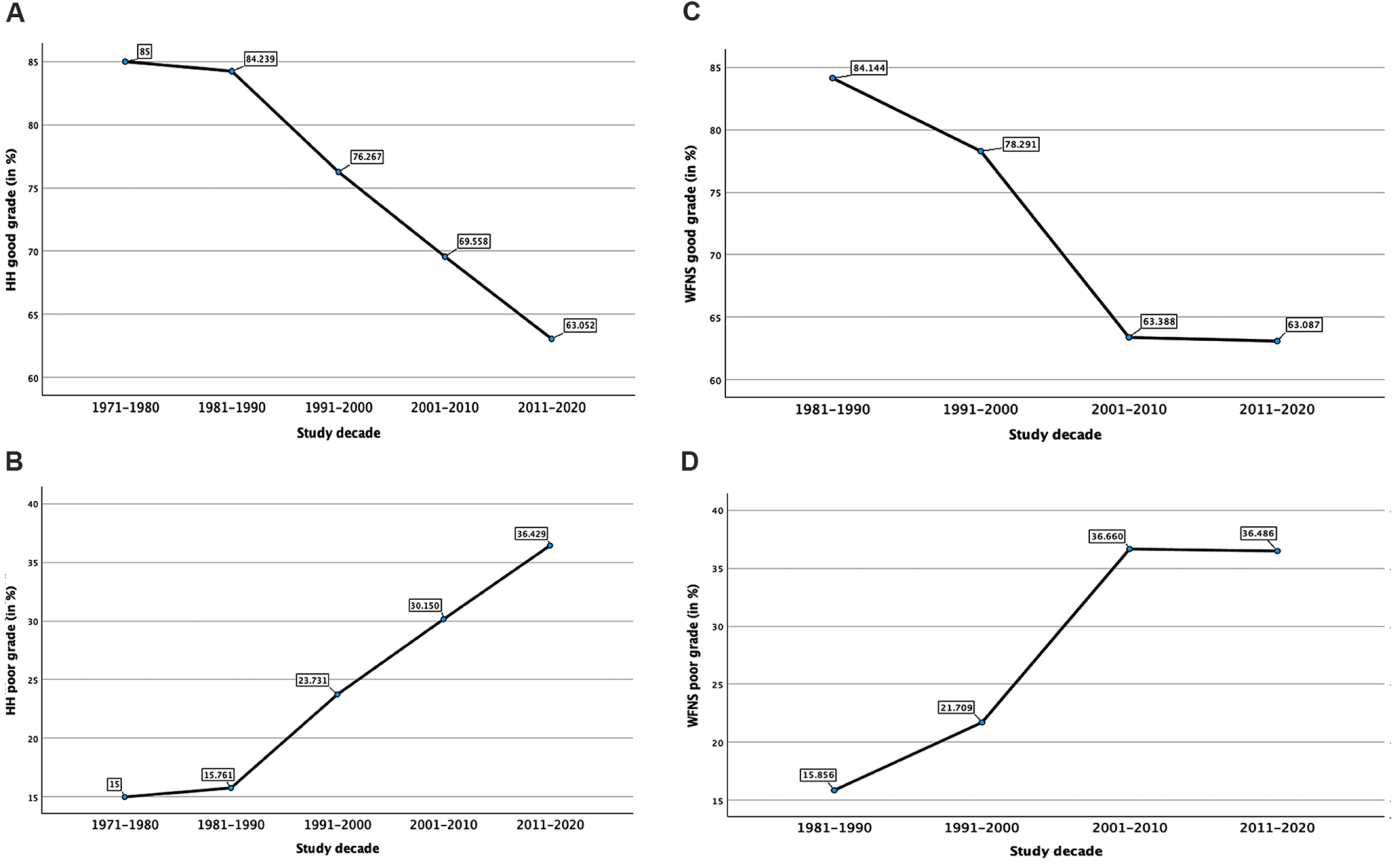
Change in aneurysmal subarachnoid hemorrhage (aSAH) grade according to the Hunt and Hess (HH) and World Federation of Neurological surgeons (WFNS) grade. **(A)** Trend of HH good grade patients from 1970–1980 until 2011–2020, an 0.741 fold decrease was observed (*p* = 0.004). **(B)** Trend of HH poor-grade patients from 1970–1980 until 2011–2020, an 2.427 fold increase (*p* = 0.004) was observed. **(C)** Trend of WFNS good grade from 1981–1990 until 2011–2020, an 0.749 fold decrease was observed (*p* < 0.001). **(D)** Trend of WFNS poor grade from 1981–1990 until 2011–2020, an 2.289 fold increase was observed (*p* < 0.001)

Throughout the decades of the study period, ANOVA analysis of the HH good grade showed a significant trend in the severity of aSAH, with the percentage of patients presenting with HH good grade decreasing by an average of 5.49% per decade (*p* = 0.004). In the decades 1970–1980, 1981–1990, 1991–2000, 2001–2010 and 2011–2020, the mean HH good grade values were 85.00%, 84.24%, 76.27%, 69.56% and 63.05%, respectively. Post-hoc analysis showed the most significant decrease from the decades 1981–1990 compared to 2011–2020 (*p* < 0.001) and from the decades 1991–2000 compared to 2011–2020 (*p* < 0.001). (Table 1)(Fig. 3A).

Concurrently, a corresponding average increase in poor grade HH scores of 5.39% per decade was observed using ANOVA analysis (*p* = 0.004). In the decades 1970–1980, 1981–1990, 1991–2000, 2001–2010, and 2011–2020 the mean HH poor grade values were 15.00%, 15.76%, 23.73%, 30.15% and 36.43%, respectively. Post-hoc analysis showed the most significant increase from the decade 1981–1990 compared to 2011–2020 (*p* < 0.001) (Table 1)(Fig. 3B).

**Fig. 4:**
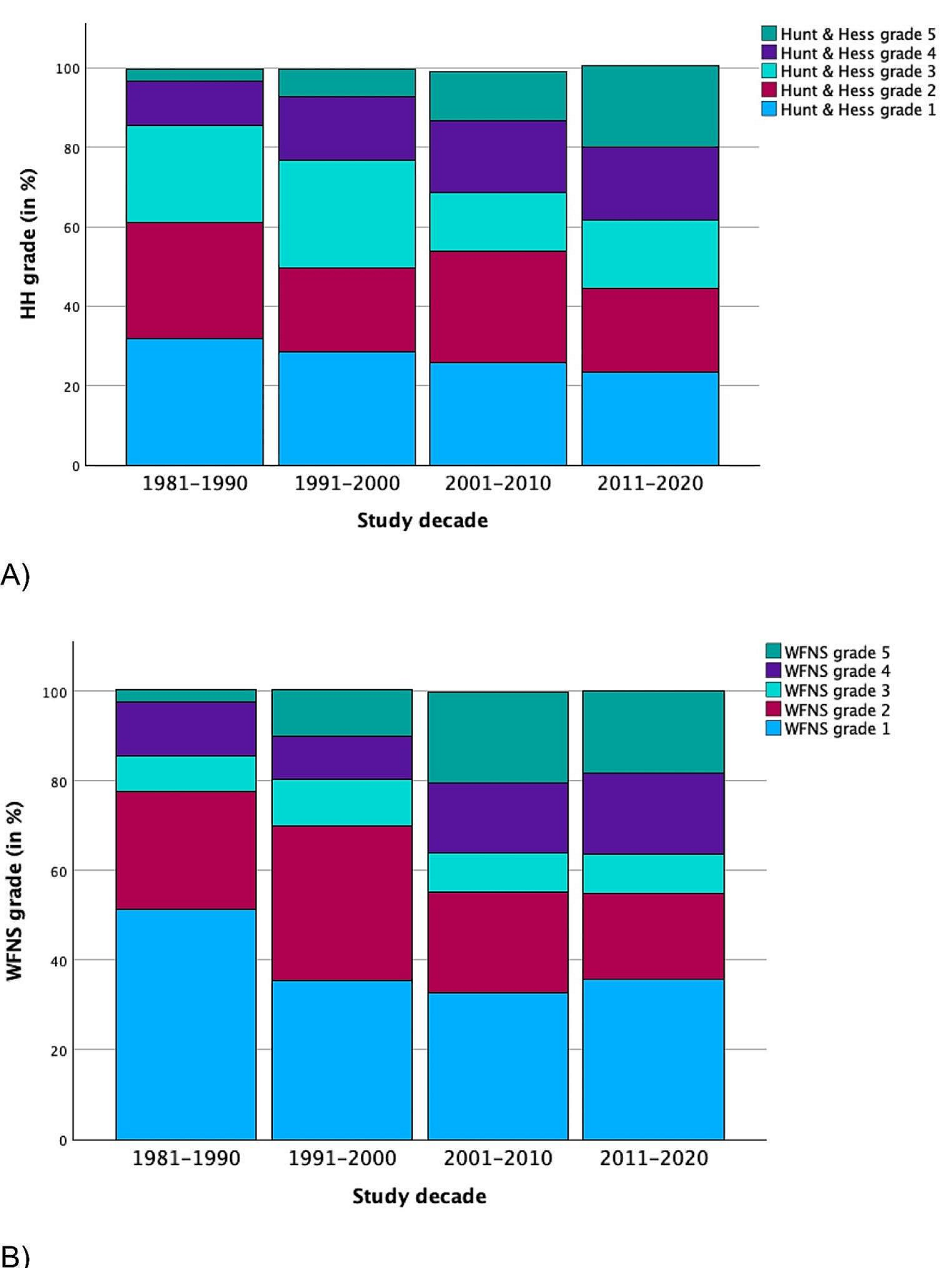
Change in single grade (1–5) aSAH according to the Hunt and Hess (HH) and World Federation of Neurological surgeons (WFNS) grade per study decade. **(A)** A significant in HH grade 5 (*p* = 0.010) was found. **(B)** A significant decrease in WFNS grade 2 (*p* = 0.010) and a significant increase in WFNS grade 4 (*p* = 0.008) and grade 5 (*p* = 0.031) were observed

ANOVA analysis of the most severe cases (HH grade 5) showed a significant increase in mean HH grade 5 per decade (*p* = 0.010) (Table 2)(Fig. 4A). In the study decades 1981–1990, 1991–2000, 2001–2010 and 2011–2020, the mean HH grade 5 values were 3.04%, 6.97%, 12.50% and 20.48%, respectively. Post-hoc analysis showed a significant increase from the decade 1981–1990 compared to 2011–2020 (*p* = 0.039). The most significant trend increase was found from the 1991–2000 to 2011–2020 (*p* = 0.032). No significant trend was observed in HH grades 1, 2, 3 and 4 (*p* = 0.798, *p* = 0.390, *p* = 0.144 and *p* = 0.315 respectively) (Table 2)(Fig. 4A).

**Table 1:** Trends in mean Grades of aSAH Patients over five decades based on Hunt and Hess (HH) and World Federation of Neurological Surgeons (WFNS) Grading Systems. The table presents the mean grade (in %) along with the standard deviation and standard error for patients classified as having good grades (1–3) and poor grades (4–5). ANOVA p-values indicate significant decreasing amount of good grade patients as well as an increasing amount of poor-grade aSAH patients

		Mean grade (in %)	Std. Deviation	Std. Error	P- Value ANOVA
HH Good grade	1970–1980	85.00	.	.	
	1981–1990	84.24	13.75	11.86	
	1991–2000	76.27	9.92	4.88	
	2001–2010	69.56	5.61	1.45	
	2011–2020	63.05	9.15	1.93	
					0.004
HH Poor grade	1970–1980	15.00	.	.	
	1981–1990	15.76	13.75	11.86	
	1991–2000	23.73	9.92	4.89	
	2001–2010	30.15	5.46	1.41	
	2011–2020	36.43	8.94	1.89	
					0.004
WFNS Good grade	1981–1990	84.14	6.47	4.39	
	1991–2000	78.29	2.81	1.15	
	2001–2010	63.39	6.94	1.55	
	2011–2020	63.09	7.38	1.65	
					< 0.001
WFNS Poor grade	1981–1990	15.86	6.47	4.39	
	1991–2000	21.71	2.81	1.15	
	2001–2010	36.66	6.98	1.55	
	2011–2020	36.49	7.30	1.63	
					< 0.001

**Table 2:** Comparative analysis of mean grades over four decades for aSAH Patients According to Hunt and Hess (HH) and World Federation of Neurosurgical Surgeons (WFNS) Grading Systems. This table outlines the mean grade (in %), standard deviation, and standard error across different grades (1–5) for both HH and WFNS classifications. The analysis employs ANOVA tests to evaluate the significance of changes over time. Notably, a decrease in WFNS grade 2 (*p* = 0.010), as well as a significant increase in HH grade 5 (*p* = 0.010) and WFNS grade 4 (*p* = 0.008) and WFNS grade 5 (*p* = 0.031) was observed

		Mean grade (in %)	Std. Deviation	Std. Error	P-value ANOVA
HH grade 1	1981–1990	31.74			
	1991–2000	28.59	13.61	7.57	
	2001–2010	25.77	9.48	2.93	
	2011–2020	23.54	10.10	2.61	
					0.798
HH grade 2	1981–1990	29.45			
	1991–2000	20.90	10.53	5.86	
	2001–2010	28.13	6.61	2.04	
	2011–2020	20.89	12.93	2.01	
					0.390
HH grade 3	1981–1990	24.20			
	1991–2000	27.11	10.58	5.88	
	2001–2010	14.63	9.91	3.06	
	2011–2020	17.31	8.84	1.55	
					0.144
HH grade 4	1981–1990	11.20			
	1991–2000	16.02	3.64	2.02	
	2001–2010	18.06	3.02	0.93	
	2011–2020	18.32	4.26	1.10	
					0.315
HH grade 5	1981–1990	3.04			
	1991–2000	6.97	5.12	2.87	
	2001–2010	12.50	5.01	1.55	
	2011–2020	20.48	9.24	2.39	
					0.010
WFNS grade 1	1981–1990	46.15			
	1991–2000	37.17	4.69	1.98	
	2001–2010	33.21	7.84	2.03	
	2011–2020	37.19	10.83	3.08	
					0.476
WFNS grade 2	1981–1990	30.65			
	1991–2000	30.11	6.20	2.62	
	2001–2010	23.09	4.98	1.29	
	2011–2020	18.42	7.48	2.13	
					0.010
WFNS grade 3	1981–1990	7.32			
	1991–2000	11.31	2.04	0.86	
	2001–2010	7.08	3.97	1.03	
	2011–2020	8.04	5.87	1.67	
					0.350
WFNS grade 4	1981–1990	13.52			
	1991–2000	9.48	1.01	0.46	
	2001–2010	16.55	3.06	0.79	
	2011–2020	16.68	5.18	1.47	
					0.008
WFNS grade 5	1981–1990	2.36			
	1991–2000	11.91	3.40	1.44	
	2001–2010	20.07	7.02	1.81	
	2011–2020	19.66	7.64	2.18	
					0.031

### Change in the world federation of neurosurgical societies grade

Similarly to the HH grade, per decade of the study period the percentage of good grade WFNS patients declined while the percentage of poor grade WFNS patients increased. ANOVA analysis showed that the percentage of patients presenting with WFNS good grade decreased by an average of 7.02% per decade (*p* < 0.001). In the study decades 1981–1990, 1991–2000, 2001–2010 and 2011–2020, the mean values were 84.14%, 78.29%, 63.39% and 63.09%, respectively. Post-hoc analysis showed a highly significant decline from the decade 1981–1990 compared to all later decades, with the most significant peak compared to the decades 2001–2010 and 2011–2020 (*p* < 0.001) (Table 1)(Fig. 3C).

Concurrently, an average increase in poor grade WFNS scores of 6.88% per decade was observed using ANOVA analysis (*p* < 0.001). In the study decade 1981–1990, 1991–2000, 2001–2010 and 2011–2020, the mean WFNS poor grade were 15.86%, 21.71%, 36.66% and 36.49%, respectively. Post-hoc analysis showed a significant increase from the decades 1981–1990 and 1991–2000 compared to 2001–2010 and 2011–2020 (*p* < 0.001) (Table 1)(Fig. 3D).

A significant decrease was observed in WFNS grade 2 patients using ANOVA analysis (*p* = 0.010). In the study decade 1980–1990, 1990–2000, 2000–2010 and 2010–2020, the mean WFNS grade 2 values were 30.65%, 30.11%, 23.90% and 18.42%, respectively (Table 2)(Fig. 4B). Post-hoc analysis showed the most significant decrease between the decade 1991–2000 compared to 2011–2020 (*p* = 0.002). Furthermore, a significant increase was observed in WFNS grade 4 patients using ANOVA analysis (*p* = 0.008). In the study decade 1981–1990, 1990–2000, 2000–2010 and 2010–2020, the mean WFNS grade 4 values were 13.52%, 9.48%, 16.55% and 16.68%, respectively. Post-hoc analysis showed the most significant increase between the decade 1991–2000 compared to 2011–2020 (*p* < 0.001). Similarly, a significant increase in WFNS grade 5 patients was observed (*p* = 0.031). In the study decade 1981–1990, 1990–2000, 2000–2010 and 2010–2020, the mean WFNS grade 5 values were 2.36%, 11.91%, 20.07% and 19.66% respectively. Post-hoc analysis showed the most significant decrease between the decades 1981–2000 and 2001–2010 (*p* < 0.001). No significant trend was found in WFNS grade 1 (*p* = 0.476) and WFNS grade 3 (*p* = 0.350).

### Secondary outcomes

Weighting the number of patients per study showed no evidence of heterogeneity.

The results of the ANOVA indicated that the differences in the number of patients among the different midyear categories were not statistically significant (*p* = 0.866).The mean number of patients per study in each decade was as follows: 223 (1970–1980), 221 (1981–1990), 410 (1990–2000), 564 (2000–2010), and 492 (2010–2020). These findings were further validated through an ANOVA analysis, which showed no significant differences in the mean number of patients between the different decades (*p* = 0.866).

Most studies originated from Europe (82 studies), followed by the United States (44 studies), Japan (27 studies) and China (27 studies)(Fig. 2). Chi square test showed no significant association between the study country and the study midyear (*p* = 0.128), percentage of HH good grade (*p* = 0.086), percentage of HH poor grade (*p* = 0.052), percentage of WFNS good grade (*p* = 0.225) and percentage of WFNS poor grade patients (*p* = 0.225).

## Discussion

This systematic review, including the most patients so far (> 100,000), shows a 2–3 fold decrease in the percentage of good grade and a 2–3 fold increase in the percentage of poor grade aSAH patients, as well as a 6–8 fold increase in grade 5 aSAH patients over the analyzed time period. This trend was specifically present in the recent decades (1991–2000 compared with 2001–2010 and 2001–2010 compared with 2011–2020) and was quantifiable using both the HH and WFNS grades. We observed this trend in all countries that contributed to this review and there were no regional differences.

What can cause a 6–8 fold increase of grade 5 aSAH patients? One possible explanation might be due to an improved emergency care system [11, 12]. On one side an improved emergency care system could allow a higher number of poor grade aSAH patients to reach the hospital alive and receive specialized treatment. On the other side it could mask the patient’s real (good) clinical status, which might be bertter, only pretending a poorer grade by early prehospital sedation and intubation. Consequently, if a patient is admitted comatose without motor responses, this could mistakenly be synononymised as solely caused by the aSAH, resulting in a HH or WFNS grade 5 [13].

It was shown for HH grade 5 aSAH patients that a reassessment of the HH grade after 48 h, results in a significant less severe grading [14]. Accordingly, it has been shown that patients with early-onset seizures were significantly more often classified as poor grade, however this group had a significantly better outcome[15]. Similar results were seen in patients graded using the WFNS grade. An assessment of the WFNS grading after resuscitation, results in a significant lower WFNS grade and a significant increase of its prognostic value [16].

The fact that the percentage of poor grade aSAH patients who achieve a favourable outcome has increased from 13% in the late 1970s and early 1980s, to 35% in the late 1980s and early 1990s, and up to 50% in the late 2020s, might, in part very well be associated with an improved medical care and the introduction of dedicated neuro-intensive care units. However, it also raises the possibility of a systematic grading error, wherein there is an increasing tendency to classify patients with an aSAH as having a poorer grade than their actual condition [7–11, 17, 18]. Therefore we suggest that consciousness and subsequently the HH or WFNS grade is (re)determined in a sedation pause to reduce this potential grading error.

The discrepancy between patients assigned to poor WFNS/HH grades and a growing proportion of favorable outcomes in this group is problematic. It shows that besides improved patient care resulting in better outcomes, also the inherent weakness of these grading systems [19]. Consequently, the unconditional withdrawal of maximal therapy for WFNS and HH grade 5 aSAH patients has become increasingly unacceptable [20–23]. Simultaneously, numerous publications show that the HH grading has a diminishing trend as a predictive parameter for the functional outcome [6, 21]. Simply put: we do need a better grading system to make meaningfull clincial decisions. Therefore, contribution of the initial HH and WFNS grading on the outcome prediction has significantly diminished over time. The historical HH and WFNS face increasing difficulties in outcome predicting in poor grade patients and is becoming less relevant with today’s improved health care [6, 7, 21, 24, 25].

The noticeable increase in the number of patients classified as poor grade, which is accompanied by a rise in favorable outcomes within this group, underscores the need to improve the specificity of grading systems by reducing the number of inaccurately classified patients and ensuring a more accurate identification of the true poor grade cases. Numerous authors have come up with propositions to improve aSAH grading. Commonly, these grades are either categorized by clinical features, radiographical features or a combination of both [23–27]. Most recent propositions lead to the incorporation of signs of brain herniation, where the poorest grade is reserved for patients with advanced signs of brainstem dysfunction such as the presence of anisocoria [13, 26, 27]. It has been shown that the inclusion of these positive signs of brainstem dysfunction, is associated with improved prediction of a poor outcome [26]. Contrary, the HH scale explicitly includes patients with (possibly early) decerebrate rigidity into grade 4, softening this possible sharp separation between grades 4 and 5 [2].

### Strengths and limitations

To our knowledge, this is the extensive systematic review of the trend of reported clinical aSAH severity in the last 5 decades. This study follows the PRISMA-P guidelines for systematic reviews, and the entire review process was conducted using the online Covidence quality control software. Two researchers used strict inclusion and exclusion criteria to decrease the risk of selection bias. Every study was judged on the amount of bias, and in concordance with three researchers (M.O, A.E.R. and C.F.), studies deemed high risk of bias using the methodological index for non-randomized studies (MINORS criteria [10]) were excluded, when the quality of evidence was deemed unacceptably low. However, despite incorporating 214 studies, the reliance on reported data in this study may introduce a source bias.

## Conclusion

This systematic review is the first study to prove a highly significant trend of an increasing amount of poor grade patients over a period of 5 decades. The higher number of poor grade aSAH patients demands an improved grading system to better differentiate grade 4 and grade 5 patients.

Registration This study was registered in the international prospective registry for systematic reviews (PROSPERO) before study eligibility selection and published on 22/04/2023 under the number “CRD42023029719” and named “Change in severity of aneurysmal Subarachnoid Hemorrhage over time: Systematic review”.

## Data Availability

No datasets were generated or analysed during the current study.

## Abbreviations

aSAH: Aneurysmal Subarachnoid Hemorrhage
ANOVA: Analysis of Variance test
HH: Hunt and Hess
WFNS: World Federation of Neurological Surgeons
